# Cost-effectiveness and budget impact of immediate antiretroviral therapy initiation for treatment of HIV infection in Côte d’Ivoire: A model-based analysis

**DOI:** 10.1371/journal.pone.0219068

**Published:** 2019-06-27

**Authors:** Eric N. Ouattara, Rachel L. MacLean, Christine Danel, Ethan D. Borre, Delphine Gabillard, Mingshu Huang, Raoul Moh, A. David Paltiel, Serge P. Eholié, Rochelle P. Walensky, Xavier Anglaret, Kenneth A. Freedberg

**Affiliations:** 1 Centre Inserm 1219, University of Bordeaux, Bordeaux, France; 2 Interdepartmental Centre of Tropical Medicine and Clinical International Health, Division of Infectious and Tropical Diseases, Department of Medicine, University Hospital Centre, Bordeaux, France; 3 Programme PAC-CI/ANRS Research Site, CHU de Treichville, Abidjan, Côte d’Ivoire; 4 Medical Practice Evaluation Center, Massachusetts General Hospital, Boston, MA, United States of America; 5 Yale School of Public Health, New Haven, CT, United States of America; 6 Division of Infectious Diseases, Massachusetts General Hospital, Boston, MA, United States of America; 7 Division of General Internal Medicine, Massachusetts General Hospital, Boston, MA, United States of America; 8 Harvard University Center for AIDS Research, Harvard Medical School, Boston, MA, United States of America; 9 Division of Infectious Disease, Brigham and Women’s Hospital, Boston, MA, United States of America; 10 Department of Health Policy and Management, Harvard T.H. Chan School of Public Health, Boston, MA, United States of America; 11 Department of Epidemiology, Boston University School of Public Health, Boston, MA, United States of America; University of Ghana College of Health Sciences, GHANA

## Abstract

**Introduction:**

The Temprano and START trials provided evidence to support early ART initiation recommendations. We projected long-term clinical and economic outcomes of immediate ART initiation in Côte d’Ivoire.

**Methods:**

We used a mathematical model to compare three potential ART initiation criteria: 1) CD4 <350/μL (*ART<350/μL*); 2) CD4 <500/*μ*L (*ART<500/μL*); and 3) ART at presentation (*Immediate ART*). Outcomes from the model included life expectancy, 10-year medical resource use, incremental cost-effectiveness ratios (ICERs) in $/year of life saved (YLS), and 5-year budget impact. We simulated people with HIV (PWH) in care (mean CD4: 259/*μ*L, SD 198/*μ*L) and transmitted cases. Key input parameters to the analysis included first-line ART efficacy (80% suppression at 6 months) and ART cost ($90/person-year). We assessed cost-effectiveness relative to Côte d’Ivoire’s 2017 *per capita* annual gross domestic product ($1,600).

**Results:**

*Immediate ART* increased life expectancy by 0.34 years compared to *ART<350/μL* and 0.17 years compared to *ART<500/μL*. *Immediate ART* resulted in 4,500 fewer 10-year transmissions per 170,000 PWH compared to *ART<350/μL*. In cost-effectiveness analysis, *Immediate ART* had a 10-year ICER of $680/YLS compared to *ART<350/μL*, ranging from cost-saving to an ICER of $1,440/YLS as transmission rates varied. *ART<500/μL* was “dominated” (an inefficient use of resources), compared with *Immediate ART*. *Immediate ART* increased the 5-year HIV care budget from $801.9M to $812.6M compared to *ART<350/μL*.

**Conclusions:**

In Côte d’Ivoire, immediate compared to later ART initiation will increase life expectancy, decrease HIV transmission, and be cost-effective over the long-term, with modest budget impact. Immediate ART initiation is an appropriate, high-value standard of care in Côte d’Ivoire and similar settings.

## Introduction

Although international guidelines recommend immediate ART initiation for persons with HIV infection, many patients in sub-Saharan Africa commonly start ART with advanced disease, when they already face high morbidity and mortality risk [1]. Timing of ART initiation has been a debate in HIV clinical care and policy, particularly in resource-limited settings [2, 3]. Multiple clinical trials over the past decade prompted the World Health Organization (WHO) to change the CD4 threshold for ART initiation, from <200 cells/*μ*L in 2006 to <350 cells/*μ*L in 2010 and then to <500 cells/*μ*L in 2013 [4–6]. In 2015, the Temprano (Côte d’Ivoire) and START (multi-country) trials showed a 44–58% reduction in mortality or serious HIV-related illnesses in people with HIV who initiated ART immediately at presentation regardless of CD4 count compared to those who deferred initiation [7, 8]. In response to these trials, WHO modified its guidelines in 2015 to recommend ART initiation at diagnosis for all people with HIV [9].

While most resource-limited countries have adopted immediate treatment, not all have fully implemented this recommendation, generally due to budget constraints and concerns about long-term affordability for all those with HIV [10]. While the Temprano and START trials showed the short-term clinical benefits of immediate ART initiation in study settings, the longer-term clinical and economic outcomes of national implementation of immediate ART initiation in Côte d’Ivoire and similar settings remain unknown. We addressed these questions by conducting a cost-effectiveness and budget impact assessment of immediate compared to delayed ART initiation in people with HIV in care and initiating care in Côte d’Ivoire.

## Methods

### Analytic overview

We used the Cost-Effectiveness of Preventing AIDS Complications (CEPAC)-International microsimulation model to assess the clinical benefits, cost-effectiveness, and budget impact of different ART initiation thresholds in a nationally representative cohort of people with HIV-1 in Côte d’Ivoire [11, 12]. We evaluated the following three ART initiation strategies: 1) CD4 count <350/*μ*L or WHO stage 3–4 (*ART<350/μL*, until 2013 the standard of care in Côte d’Ivoire); 2) CD4 count <500/*μ*L or WHO stage 3–4 (*ART<500/μL*, the standard of care in Côte d’Ivoire until April, 2017) [10]; or 3) ART initiation when patients present to care, regardless of CD4 count (*Immediate ART)*, the current WHO recommendation and recent guideline in Côte d’Ivoire [9, 13]. We simulated these strategies for the population of people with HIV in care in Côte d’Ivoire.

We projected several outcomes: life expectancy from time of presentation to care; cumulative 10-year HIV transmissions; 5- and 10-year total medical resource use; 10-year cumulative life-years saved; and 10-year incremental cost-effectiveness ratios (ICERs) in 2017 US dollars per year of life saved (YLS). To assess budget impact, we projected 5- and 10-year HIV-related expenditures for *Immediate ART* compared to *ART<500/μL* and *ART<350/μL* for people with HIV in care and those projected to initiate ART from 2017–2021 in Côte d’Ivoire. Cost-effectiveness and budget impact also accounted for transmitted cases of HIV. When reported for purposes of economic evaluation, all outcomes were discounted at a rate of 3% per year. In accordance with convention, budget impact results were reported undiscounted [14]. We defined a strategy as cost-effective if its ICER was less than $1,600/YLS, the 2017 annual *per capita* GDP in Côte d’Ivoire [15].

### Cohort description

#### Population included in the cost-effectiveness analysis

For the cost-effectiveness analysis, we began by modeling the 170,000 persons currently in care in Côte d’Ivoire, as well as all incident transmitted cases from this cohort (first-generation and later-generation transmissions over 10 years, S1A Fig). Incident cases began unlinked to care, with characteristics of a newly-infected population, and presented to care through routine HIV screening or clinical presentation with an opportunistic disease (OD) [16].

#### Population included in the budget impact analysis

For the budget impact analysis, we included HIV-related expenditures incurred by all those in care and entering care in the next 5 years in Côte d’Ivoire. To estimate the number expected to enter care each year (the “present to care” cohort), we began with historical data showing a yearly average of 14,000 people entering HIV care in Côte d’Ivoire [17]. For the *Immediate ART* and the *ART<500/μL* strategies, we subtracted the number of transmissions prevented compared with the *ART<350/μL* strategy from 14,000 to estimate the “present to care” cohort size (S1B Fig).

### The CEPAC-International model

#### Disease model

CEPAC-International is a microsimulation model of HIV natural history, disease progression, and treatment [12, 18]. Simulated patients are followed monthly from model entry until death and are generated from user-specified distributions of initial age, sex, initial CD4 count, HIV RNA, treatment adherence, and OD history. People with HIV who enter the model not in care can link to care through presentation with an OD or a monthly probability of having an HIV test and presenting to care. ART is initiated according to strategy-specific CD4 count thresholds or based on an OD. Effective ART reduces HIV RNA and increases CD4 count; CD4 count determines risk of ODs and death [19, 20]. Additional details of the model are published and online at http://web2.research.partners.org/cepac [18, 21, 22].

#### Transmission model

We modeled HIV transmission dependent on stage of infection (acute, chronic, or end-stage disease) and HIV RNA [23]. The model reports monthly, patient-level HIV RNA; this depends on baseline HIV RNA, stage of infection, and virologic suppression on ART. Summing cumulative viral load across 12 months and linking this to HIV RNA-specific transmission rates determines yearly new infections [23]. In this way, we account for transmissions from the “first generation” cohort, or transmissions from patients that are simulated from model initiation. We also use this method to calculate transmissions from each previous year’s newly-infected cohort for up to 10 years. Outcomes in each subsequent year include the first-generation cohort as well as all newly infected cohorts from previous years (S1A Fig).

### Model input data

#### Cohort characteristics and natural history

Simulated people with HIV in care in Côte d’Ivoire had a mean initial CD4 count of 259/*μ*L (SD: 198/*μ*L) (Table 1). Age and sex distributions were from the PRECO-CI cohort [16], a nationally representative cohort of people with HIV in care in Côte d’Ivoire, where the majority of people in care have CD4 counts <500/*μ*L (88%). Initial HIV RNA distribution was from the Temprano trial [7]. The incidence of tuberculosis (TB) for people with HIV at CD4 counts >350/*μ*L was also derived from the Temprano trial, which closely captured TB incidence data at high CD4 counts [7, 19]. Other natural history characteristics were from published cohort studies of the HIV-infected population in Côte d’Ivoire [19, 24–26]. Transmitted cases began the simulation with characteristics of a newly-infected cohort (high CD4 count, no prior OD history) and were diagnosed and linked to care, with subsequent treatment as described below, at a mean CD4 count of 259 cells/*μ*L based on current HIV testing and presentation data [16].

**Table 1:** Main input parameters for an analysis of the cost-effectiveness of immediate ART for HIV infection in Côte d’Ivoire.

Parameter	Base-case value	Range evaluated	Reference
**Cohort characteristics**			
Sex, female/male, %	68/32	—	[16]
Age, mean (SD) years	37 (9)	18–55	[16]
CD4, mean (SD) cells/*μ*l (Total Cohort)	259 (198)	146–388	[16]
Plasma HIV-1 RNA distribution, copies/mL %			[7]
>100,000	34	—	
30,001–10,0000	24	—	
10,001–30,000	17	—	
3,001–10,000	13	—	
501–3,000	8	—	
50–500	3	—	
< 49	1	—	
**Monthly probability of morbidity and mortality, off ART, % §**			
Monthly OD rates (non-TB)	0.01–9.0	—	[19, 27]
Monthly TB rates	0.2–0.7	—	[7, 27]
Acute mortality from OD	0.0–16.7	—	[19, 27]
Acute mortality from TB	6.5–50.0	—	[19, 27]
Monthly probability of death from HIV	0.04–5.4	—	[27]
**ART efficacy, toxicity, and loss to follow-up**		
First-line ART*			
HIV-1 RNA suppression at 6 months, mean %	80	50–90	[25]
Virologic failure after 6 months, per 100 PY	15	7–22	[25]
Adherence <65%**	93	—	[25]
Adherence >95%	1.6	—	[25]
Monthly CD4 increase for those suppressed, mean (SD) cell/*μ*l			
Between 0 and 2 months	76 (19)		[25]
≥ 3 months	4 (1)	—	[25]
Toxicity, %			
Minor	11	—	[28]
Major	5	—	[29]
Toxicity-related mortality, %	0.6	—	[28, 29]
Loss to follow-up, per 100 PY			[30, 31]
Adherence <65%	13	6–17	
Adherence >95%	1.9	5–14	
**Transmission rates (per 100 PY), by disease stage and viral load**			[23, 32]
Incident infection (6 months post infection)^†^	65.47	28.06–152.90	
Late stage disease (CD4 <200 cells/*μ*L)	9.03	3.87–21.09	
>100,000 copies/mL	9.03	3.87–21.09	
10,001–100,000 copies/mL	8.12	2.78–23.77	
3,001–10,000 copies/mL	4.17	0.84–20.65	
501–3,000 copies/mL	2.06	0.57–7.47	
0–500 copies/mL	0.16	0.02–1.13	
** Costs, 2017 USD**			
ART, annual			[33, 34]
1^st^-line ART	90	45–135	
2^nd^-line ART	282	141–423	
Prophylaxis, annual			
Co-trimoxazole	27	—	[18]
Laboratory monitoring, per test			
CD4 test	13	—	CeDReS
HIV RNA test	40	—	CeDReS
OD treatment costs (range by OD)	80–555		[18]

ART: antiretroviral therapy; SD: standard deviation; USD: 2017 US dollars; PY: person-years; CeDReS: Centre de diagnostic et de Recherche sur le SIDA et les Affections Opportunistes at Treichville University Hospital, Abidjan, Côte d’Ivoire; OD: opportunistic disease

§ Morbidity and mortality ranges in patients from higher to lower mean CD4 strata

*1st-line ART: tenofovir + lamivudine + efavirenz

**Based on Medication Possession Ratio [25]

†The transmission rate for incident infection is derived as 7.25*9.03 [23, 32]

#### ART regimen

Overall six-month virologic suppression was 80% for both 1^st^- line (tenofovir and lamivudine plus efavirenz) and 2^nd^-line ART (zidovudine and lamivudine plus lopinavir/ritonavir) regimens. This reflects current 1^st^-line ART and levels of virologic suppression in clinical settings in Côte d’Ivoire [25]. Probability of later failure after initial suppression was 0.13–9.0%/month, depending on adherence [12]. After failure on either regimen, patients received an adherence intervention, with a 54% probability of viral resuppression, consistent with current approaches to management [35]. Guideline-concordant care for Côte d’Ivoire included twice-yearly CD4 tests, with HIV RNA confirmation of CD4-defined treatment failure [6, 13].

#### Transmission

Aggregated transmission probabilities by HIV RNA stratum ranged from 0.16/100PY for HIV RNA<500 copies/mL to 9.03/100PY for HIV RNA>100,000 copies/mL [23]. Transmission was increased during the 6-month period of acute HIV infection (65.47/100PY) and during end-stage disease off ART with CD4<200/*μ*L (9.03/100PY) [32].

#### Cost inputs

Cost inputs to the model included all direct HIV-related medical costs. ART costs were based on Clinton Health Access Initiative prices and included $90/person/year for first-line ART and $282/person/year for second-line therapy [33, 34]. OD treatment costs ranged from $80-$555 and CD4-stratified care costs from Côte d’Ivoire ranged from $37-$50/month [36]. We used Côte d’Ivoire-specific GDP deflators and the average 2017 exchange rate to convert all cost inputs to constant 2017 US dollars (USD) [15]. The analysis was conducted from a modified societal perspective (not including patient time costs).

### Sensitivity analyses

We assessed key input parameters in sensitivity analysis to evaluate the robustness of our findings to plausible variation in these parameters (ranges in Table 1). Combining influential parameters identified in one-way sensitivity analyses, we simultaneously varied multiple input parameters to determine their combined impact on the results. In the base-case analysis, we assumed no effect of changing ART initiation criteria on overall HIV testing rates. We tested this assumption in sensitivity analysis by increasing the mean CD4 count at diagnosis for new infections, as might occur if HIV testing rates increased.

### Ethics statement

The CEPAC Data Repository was approved by the Massachusetts General Hospital IRB (2014P002708/MGH). Within the CEPAC Data Repository, all data are collected by outside entities and shared with investigators after a quality check by a compliance specialist to ensure that they meet all specifications of the Data Use Agreement and that all identifiers are removed. The Temprano data set and consent form were included in the CEPAC Data Repository at the time of the initial approval of the protocol. The Temprano trial protocol was approved by the Côte d’Ivoire Minister of Health and Public Hygiene and the Côte d’Ivoire National Ethics Committee for Life Sciences and Health (/MSHP/CAB/CNESVS/06). The Temprano dataset as received from the collaborators in Côte d’Ivoire did not contain any Protected Health Information. The dataset as received contained limited indirect identifiers necessary for the analyses, but it was stored in a restricted secure network drive to which only named CEPAC investigators and biostatisticians have access. The biostatistical team generated completely anonymized aggregate inputs to be used in the model, which were then made available to the larger study team. As the research project was limited to use of existing secondary data provided by the Temprano investigators in a de-identified format, the Partners Human Research Committee waived the requirement for informed consent.

## Results

### Clinical outcomes

Discounted life expectancy from presentation to care increased from 16.05 years with *ART<350/μL* to 16.22 with ART at 500/*μ*L and to 16.39 years with *Immediate ART* (Table 2A). Cumulative transmissions arising from the 170,000 persons in care and their transmitted cases over 10 years decreased from 47,500 with *ART<350/μL* to 43,000 with *Immediate ART*, a decrease of 9.8% (Table 2A and Fig 3 bottom). Because most people in care in Côte d’Ivoire have a CD4 count <350/*μ*L, there were 22,780 people with CD4 counts 350-500/*μ*L and 20,400 people with CD4 counts >500/*μ*L at simulation start; thus the *Immediate ART* strategy changed care in 12% of the in-care population. When we included life-years saved from transmissions averted over 10 years and compared total life-years to *ART<350/μL*, *ART<500/μL* saved 12,000 years of life and *Immediate ART* saved an additional 4,000 years of life (both discounted).

**Table 2:** Clinical and economic outcomes of ART initiation according to CD4 threshold or immediate ART initiation in Côte d’Ivoire.

Transmissions and cost-effectiveness*						
	**Life expectancy (years)** ^†^	**Transmissions caused, 10y**	**Total life-years,** **10y**	**Total costs,** **10y**		**ICER, 10y ($/YLS)**
**Strategy**						
ART<350/*μ*L	16.05	47,500	1,379,000	1,056,630,000		-
ART<500/*μ*L	16.22	44,800	1,391,000	1,064,610,000		Dominated**
Immediate ART	16.39	43,000	1,395,000	1,067,610,000		680
**5-year budget impact, 2017 USD, in millions**^‡^						
	**ART costs (millions USD)**	**Lab monitoring costs (millions USD)**	**Other HIV care costs (millions USD)**	**Total costs** **(millions USD)**	**Increase in expenditures compared to ART<350/*μ*L, (%)**	
**Strategy**						
ART<350/*μ*L	85.5	40.2	676.3	801.9	-	
ART<500/*μ*L	91.2	41.0	676.7	809.0	7.1 (0.88)	
Immediate ART	94.8	41.6	676.2	812.6	10.7 (1.33)	

y: year; ICER: incremental cost-effectiveness ratio; ART: antiretroviral therapy; YLS: year of life saved.

*Results in panel A (except for transmission outcomes) are discounted at 3% per year. The results are for the prevalent cohort of 170,000 people with HIV in care in Côte d’Ivoire and their transmitted cases. Life-years and costs are rounded after calculating the ICER.

†Life expectancy is reported from time at entry to care.

‡Results in panel B are undiscounted by convention [14]. The results are for the prevalent cohort of 170,000 people with HIV in care in Côte d’Ivoire, their transmitted cases, and the additional estimated number of persons entering HIV care each year.

**Dominated: A strategy that is less cost-effective (higher ICER) than the next most costly option, and thus not an economically efficient use of resources [14]. The ICER for ART<500/*μ*L compared to ART<350/*μ*L is $680/YLS.

### Costs and cost-effectiveness analysis

Over 10 years, total discounted costs increased from $1.057 billion with *ART<350/μL* to $1.065 billion for *ART<500/μL* and to $1.068 billion with *Immediate ART*. Compared to *ART<350/μL*, *Immediate ART* was cost-effective with an ICER of $680/YLS, well below the annual *per capita* GDP in Côte d’Ivoire (Table 2, undiscounted results in S1 Table). *ART<500/μL* had a higher ICER than *Immediate ART* (ICER $680/YLS compared to *ART<350/μL*) and by convention, we labeled this strategy “dominated,” or an inefficient use of economic resources.

### Sensitivity analyses

#### One-way sensitivity analyses

**Fig 1:**
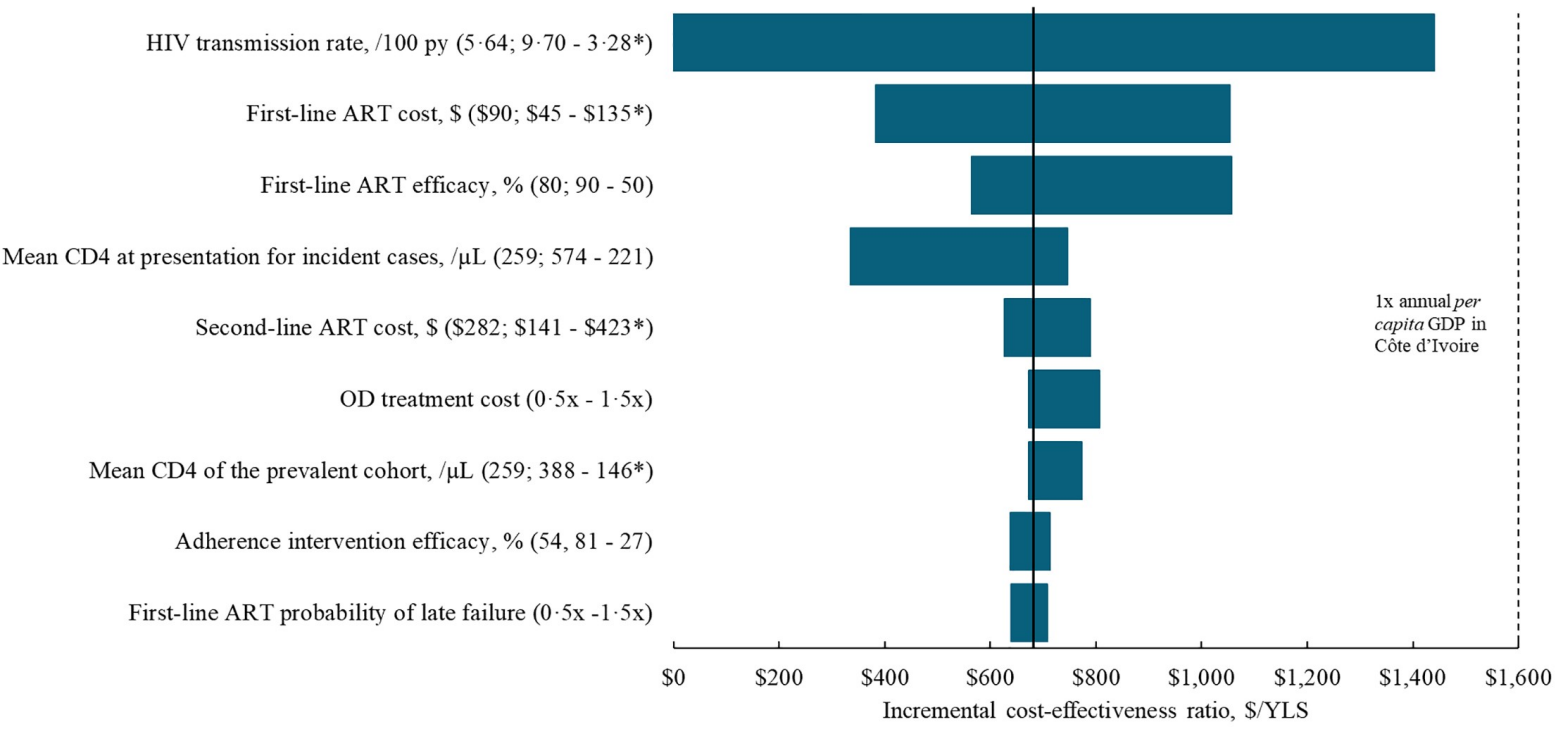
One-way sensitivity analysis, *Immediate ART* strategy compared to *ART<350/μL*. This tornado diagram depicts the impact of uncertainty in various input parameter ranges on the incremental cost-effectiveness ratio (ICER) of *Immediate ART* compared to *ART<350/μL*, except for asterisked bounds (*), which are compared to *ART<500/μL*. The bold black vertical line crosses the horizontal axis at the base-case ICER of $680/YLS. Each bar represents the effect of changing one input parameter across its plausible range, and plotting the resulting ICERs of *Immediate ART* compared to *ART<350/μL* (or to *ART<500/ μL* in cases where *Immediate ART* does not display extended dominance compared to *ART<500/ μL*; see Methods for details). Along the vertical axis, the parameter varied is reported as (Base-case value; value leading to lower ICER-value leading to higher ICER). No changes in individual parameters increase the ICER to over the 2017 annual *per capita* GDP in Côte d’Ivoire of $1,600 (dashed vertical line). ART: antiretroviral therapy; YLS: years of life saved; GDP: annual *per capita* gross domestic product. OD: opportunistic disease.

In one-way sensitivity analyses, with variation across plausible input ranges, the ICER for *Immediate ART* compared to *ART<350/μL*, as well as to *ART<500/μL*, remained below the annual Côte d’Ivoire *per capita* GDP (Fig 1). *ART<500/μL* was no longer a “dominated” strategy if costs of 1^st^- and 2^nd^-line ART were higher, mean CD4 count at diagnosis for the prevalent cohort was lower, or HIV transmission rates were lower (* in Fig 1). Compared to *ART<350/μL*, the cost-effectiveness of *Immediate ART* was most sensitive to HIV transmission rates stratified by HIV RNA level, first-line ART cost, and first-line ART efficacy at presentation for incident cases.

When we varied transmission rates across their literature-based 95% CI (3.28–9.70/100PY), *Immediate ART* ranged from cost-saving to an ICER of $1,440/YLS; with higher transmission rates *Immediate ART* was more cost-effective. When 1^st^-line ART costs were varied from $45-135/year (base-case: $90/year), the ICER ranged from $380/YLS to $1,050/YLS. *Immediate ART* became more cost-effective (ICER $330/YLS) with higher CD4 count at diagnosis for incident cases (574 cells/*μ*L), as might be the case with increased testing, and less cost-effective (ICER $750/YLS) with lower CD4 count at diagnosis (221 cells/*μ*L), if testing were to decrease.

#### Multi-way sensitivity analyses

**Fig 2:**
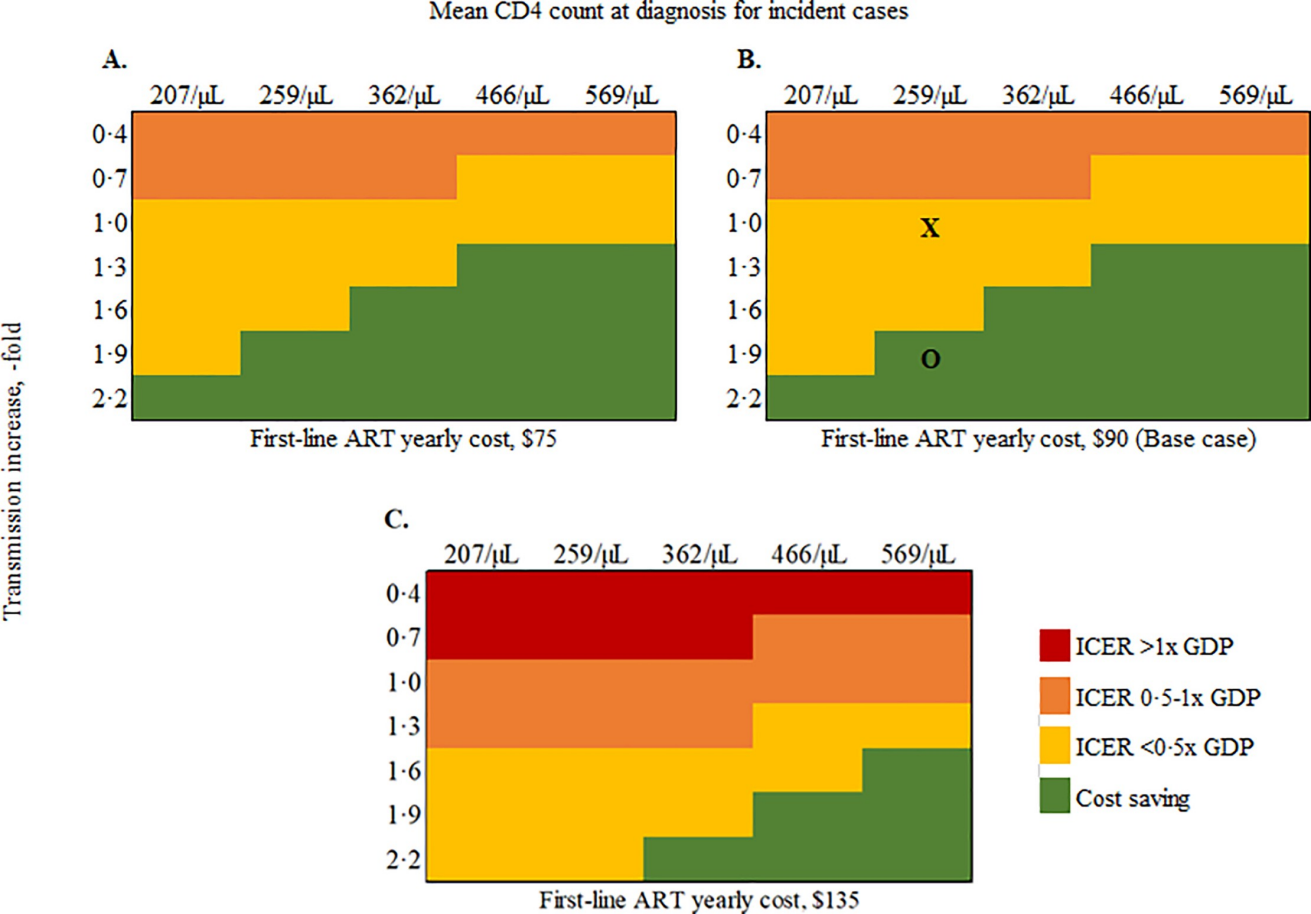
Multi-way sensitivity analysis on transmission rate, CD4 count at diagnosis, and ART costs in a model-based analysis of Immediate ART initiation in Côte d’Ivoire. This shows the impact of uncertainty in three key input parameters on the incremental cost-effectiveness ratio (ICER) of *Immediate ART* compared to *ART<350/μL*. We varied transmission rates across reported 95% confidence intervals on the vertical axis. On the horizontal axis, we varied the CD4 count at which transmitted cases present to care. Panel A represents a yearly first-line ART cost of $75. Panel B represents base-case ART costs. The base-case combination of input parameters is marked with the **X**. The parameter combination at which *Immediate ART* becomes cost-saving is marked with an **O**. Panel C represents yearly first-line ART costs that are 1·5x base-case values ($135). Combinations of the above parameters that resulted in ICERs that are cost-saving are in green, <0·5x the Côte d’Ivoire annual *per capita* GDP ($1,600) in yellow, 0·5-1x GDP in orange, and >1x GDP in red. ART: antiretroviral therapy; GDP: annual *per capita* gross domestic product.

We simultaneously varied transmission rates, mean CD4 count at diagnosis for incident cases, and first-line ART cost across wide ranges (Fig 2). When we held each of these at their base-case values, increasing transmission rates 1.9-fold or more made *Immediate ART* cost-saving (Fig 2, panel B, **O**). If the cost of first-line ART was lowered to $75/year, no combination of input parameter variation resulted in an ICER for *Immediate ART* above $1,600, Côte d’Ivoire’s 2017 annual *per capita* GDP (Fig 2, panel A). Only if ART costs increased 1.5-fold and transmission rates decreased to less than 0.7-fold did the ICER for *Immediate ART* rise above the Côte d’Ivoire annual *per capita* GDP (Fig 2, Panel C).

### Transmissions and budget impact

At 5 years, *ART<500/μL* and *Immediate ART* increased the budget compared to *ART<350/μL* by 0.88% and 1.33%, from $801.9 million to $809.0 million and $812.6 million (undiscounted). The annual expenditures over the first 5 years for *ART<500/μL* compared to ART<*350/μL* ranged from $1.0 million to $2.5 million and for *Immediate ART* compared to ART<*350/μL* ranged from $1.4 million to $4.4 million (S4 Table). In the *Immediate ART* strategy at 5 years, laboratory monitoring costs increased by 0.2% and ART costs increased by 1.2% compared to *ART<350/μL*; other HIV care costs did not change substantially (S2 Fig).

**Fig 3:**
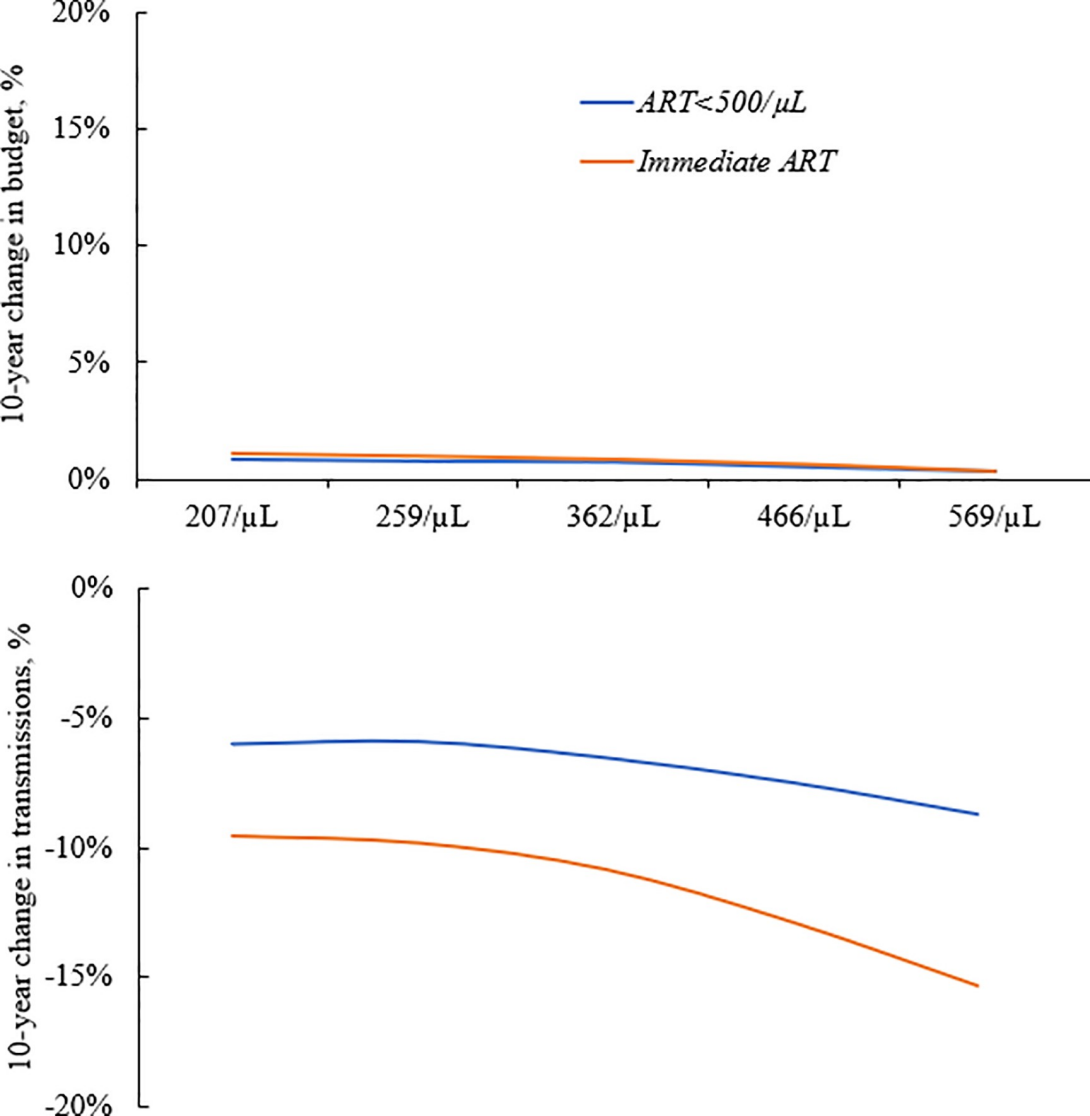
HIV care budget increase and reduction in transmissions by ART initiation strategy and CD4 count at presentation to care, both after 10 years. The 10-year change in budget, top, and 10-year change in transmission, bottom, for the *ART<500/μL* (blue) and *Immediate ART* (orange) strategies compared to *ART<350/μL*, is shown by CD4 count at presentation to care ranging from 207/*μ*L to 569/*μ*L (base-case 259/*μ*L). *ART<500/μL* and *Immediate ART* proportionally reduce transmissions substantially more than they increase the budget at 10 years. If incident cases present with higher CD4 counts, due to increased testing and outreach, the reduction in transmission is even greater. ART: antiretroviral therapy.

Delayed ART initiation increases both morbidity and mortality, as observed by the greater number of life-years gained from immediate ART initiation compared with delayed initiation (*ART<500/μL*) across initial CD4 counts (S2 Table). As the mean CD4 count at presentation to care of the incident cohorts increases, newly-infected patients spend more time in care and waiting to meet treatment criteria with the delayed ART strategies, while at risk of developing ODs and incurring additional treatment costs. Thus, the total costs of A*RT<500/μL* approach and then exceed the total costs of *Immediate ART* as the mean CD4 counts of the incident cohorts increase, as would likely be seen with increased testing (S2 Table). Over 10 years, the *Immediate ART* strategy also had a larger impact on decreasing transmissions than on increasing the budget; these differences became even more pronounced as the CD4 count at diagnosis increased (Fig 3).

## Discussion

We assessed the clinical impact, cost-effectiveness, and budget impact of immediate ART initiation for all people with HIV currently in care in Côte d’Ivoire, regardless of CD4 count, as well as those presenting to HIV care over the next 5 years. The latest HIV treatment guidelines in Côte d’Ivoire recommend immediate ART for all people with HIV; this analysis used nationally representative data to evaluate this guideline change. Our analysis had three main findings.

First, compared to *ART<350/μL*, *Immediate ART* improved overall discounted life expectancy from presentation to care (from 16.05 years to 16.39 years) and reduced HIV transmissions over 10 years (from 47,500 to 43,000). Second, *Immediate ART* is cost-effective compared to both *ART<350/μL* and *ART<500/μL*. While the increases in life expectancy are relatively small, particularly when averaged across all persons already in care, the increases in costs are also small, since most people with HIV in care in Côte d’Ivoire are already on ART, and *Immediate ART* will prevent additional transmissions, thus avoiding future care costs. When we examined the 170,000 people with HIV in care in Côte d’Ivoire, and their transmitted cases, the incremental cost-effectiveness ratio for *Immediate ART* compared to *ART<350/μL* was $680/YLS, less than half the *per capita* GDP in Côte d’Ivoire ($1,600). Third, compared to *ART<350μL*, *Immediate ART* increased the 5-year HIV budget in Côte d’Ivoire by 1.3%, a modest increase that accounted for current patients, as well as those newly-diagnosed and likely to enter care during the subsequent five years.

Our cost-effectiveness conclusions were robust to wide variations in key clinical characteristics and costs. When single parameters were varied, the ICER of *Immediate ART* never exceeded the *per capita* GDP in Côte d’Ivoire. In multiway sensitivity analyses, the ICER of *Immediate ART* remained below $1,600/YLS for plausible combinations of transmission rates, CD4 counts at diagnosis for incident cases, and first-line ART efficacies. Only if ART costs increased by 1.5-fold did the ICER begin to exceed the Côte d’Ivoire *per capita* GDP. However, as ART costs in resource-limited settings have historically decreased over time, this is unlikely. Further, even new integrase-inhibitor based regimens are now being priced at $75/person/year in many resource-limited settings [37]; if this occurs in Côte d’Ivoire *Immediate ART* would be even more cost-effective. Finally, if we examine a longer time horizon than 10 years, *Immediate ART* becomes even more cost-effective. At 15 years, the ICER for *Immediate ART* compared to *ART<350/μl* decreases to $330/YLS (from $680/YLS at 10 years); at 20 years, the ICER for *Immediate ART* further decreases to $250/YLS.

This study adds to the growing literature demonstrating the clinical benefit and cost-effectiveness of earlier ART initiation among HIV-infected individuals [38, 39]. Similar to our findings, they found immediate ART to be cost-effective or cost-saving. Kuznik et al. used a Markov model to examine immediate ART in South Africa, Nigeria, Uganda, and India, and found that immediate ART would be either cost-saving or cost-effective using a 1X per capita GDP threshold [40]. McCreesh et al. similarly found immediate ART to be cost-effective by similar criteria in a model focused in Uganda [41]. Our analysis also complements the clinical findings of the Temprano trial. We demonstrate additional projected long-term clinical benefit and economic value to *Immediate ART* compared to *ART<500/μL*.

A major concern regarding immediate ART provision is the budget impact associated with treating all patients. We found that the 5-year budget impact of *Immediate ART* as a strategy compared to *ART<350/μL* is modest in the context of the overall HIV program in Côte d’Ivoire (1.3% increase, or $10.7M). This is because many people with CD4 counts >350/*μ*L in Côte d’Ivoire, and elsewhere, have not yet been tested for HIV and are not in care; additionally, most of those currently tested do not have CD4 counts >500/*μ*L. If *Immediate ART* were combined with increased HIV testing efforts, resulting in a higher mean CD4 count at linkage to care, we found, however, that the absolute budget impact of *Immediate ART* would be even less. This is due to savings from preventing opportunistic diseases, such as tuberculosis and bacterial infections, which are the most common complications in Côte d’Ivoire and similar settings in people with higher CD4 counts, as well as preventing HIV transmissions. In fact, recent evidence suggests that increasing ART availability may itself increase the number of patients seeking HIV testing, even without any testing-specific interventions [42].

This analysis has several limitations. First, simulation models necessarily simplify complex biological processes and procedures, and not all data are available from a single setting. We used transmission rates from published meta-analyses, rather than specifically from Côte d’Ivoire. We found, however, that the cost-effectiveness conclusions were robust to variations in these rates. Second, we derived clinical care costs from a single setting in Côte d’Ivoire. The results did not change even when we varied these costs widely. Third, we did not specifically account for NNRTI resistance in the model. The immediate ART strategy, however, remained cost-effective even with lower ART efficacy, as might be found with primary drug resistance. In the cost-effectiveness analysis, we examined outcomes only for individuals in HIV care and their transmitted cases. As a result, our transmission projections do not include those occurring from people not in care. There was no material change in the qualitative findings and conclusions of the cost-effectiveness analysis when we varied the number of transmissions included. We also did not adjust for health-related quality of life in this analysis. Since additional years lived will be in less than perfect health, our costs per year of life saved would be lower than cost per quality-adjusted life-year (QALY) or disability-adjusted life-year. However, if immediate ART increases quality of life in addition to improving survival, our costs per year of life saved would be higher than cost per QALY. It is also not clear that there is a strong relationship between CD4 count and health-related quality of life in the current era of effective ART [43, 44]. Thus, readers should interpret our comparison to the WHO-CHOICE GDP threshold with caution. Finally, we did not specifically include the cost of any increased testing, which would further increase the number of people in care in Côte d’Ivoire. However, we and others have shown that HIV testing itself is highly cost-effective in multiple settings, including those with both higher and lower prevalence than in Côte d’Ivoire [45–48]. In this analysis, even if increased testing increased the CD4 count at presentation, *Immediate ART* would remain cost-effective. As the number of people in care increased, total outlays would also increase.

The pressing question of ‘when to treat’ HIV-infected individuals in resource-limited settings has been answered in recent clinical trials, with immediate ART treatment demonstrating clear short-term clinical superiority over delayed treatment strategies [6–8]. In this analysis, we aimed to assess the longer-term clinical and economic implications of immediate ART initiation, focusing on Côte d’Ivoire, a nation with high HIV prevalence in West Africa. We found that ART initiation at presentation would increase life expectancy, decrease HIV transmissions, and be highly cost-effective, all with a modest budget impact over 5 years. Immediate ART is an appropriate standard of care in Côte d’Ivoire and similar countries in sub-Saharan Africa and should be fully implemented.

## Supporting information

S1 Fig Populations modeled in the cost-effectiveness and budgetary impact analyses of a model-based analysis of early ART initiation in Côte d’Ivoire. The populations included in the cost-effectiveness analysis (Panel A) were the 170,000 persons currently in care in Côte d’Ivoire (CI) as well as all transmitted cases arising from this population (including first generation and higher order transmissions). We restricted the population modeled to persons in care, and their transmitted cases, excluding persons with undiagnosed HIV, to isolate the effect of a policy change regarding ART initiation criteria. The populations modeled in the budget impact analysis (Panel B) included the 170,000 persons currently in care in Côte d’Ivoire and their transmitted cases, as well as persons newly presenting to care each year over the next five years. Each outlined box represents the entry of the specified cohort into the analysis. To estimate the number expected to enter care each year over the next 5 years (the “present to care” cohort), we began with historical data showing a yearly average of 14,000 people entering HIV care in Côte d’Ivoire [17]. For the *Immediate ART* and the *ART<500/μL* strategies, we subtracted from the 14,000 the number of transmissions prevented compared with the *ART<350/μL* strategy. Because the *ART<350/μ*L cohort has the most transmissions of the strategies modeled, any transmissions averted by *ART<500/μL* and *Immediate ART* are excluded in the budget impact analysis for those strategies. We included an estimate of the costs of undiagnosed persons presenting to care in Côte d’Ivoire in the budget impact analysis to better project total HIV program costs under the different ART initiation thresholds.
(TIF)

S2 Fig 5-year budget impact of ART at CD4 counts <500/μL and immediate ART initiation compared to ART at CD4 counts <350/μL in Côte d’Ivoire. Each bar represents the 5-year proportional budget impact of *ART<500/μL* (left) and *Immediate ART* (right) compared to *ART<350/μL*. The height of the bars represents the impact on the total budget, measured in % budget increase compared to *ART<350/μL*. The change in other HIV care costs (orange), laboratory monitoring costs (blue), and ART costs (green) are also shown as a proportion of the change in total costs compared to *ART<350/μL* Most of the budget increases for *ART<500/μL* and *Immediate ART* at 5 years are in ART costs. **ART**: antiretroviral therapy.
(TIF)

S1 Table Undiscounted clinical and economic outcomes of ART initiation according to CD4 threshold or immediate ART initiation in Côte d’Ivoire, corollary to Table 2. (DOCX)

S2 Table Sensitivity analysis of mean CD4 at diagnosis for incident cohorts in evaluation of clinical and economic outcomes of ART initiation according to CD4 threshold or immediate ART initiation in Côte d’Ivoire. (DOCX)

S3 Table 15- and 20-year clinical and economic outcomes of ART initiation according to CD4 threshold or immediate ART initiation in Côte d’Ivoire. (DOCX)

S4 Table 5-year annual budget impact, 2017 USD, in millions. (DOCX)
