# Supplementary figures and images for: Cost-effectiveness and budget impact of immediate antiretroviral therapy initiation for treatment of HIV infection in Côte d’Ivoire: A model-based analysis
Source: PLoS One. 2019 Jun 27;14(6):e0219068. doi: 10.1371/journal.pone.0219068 (PMC6597104; doi:10.1371/journal.pone.0219068)

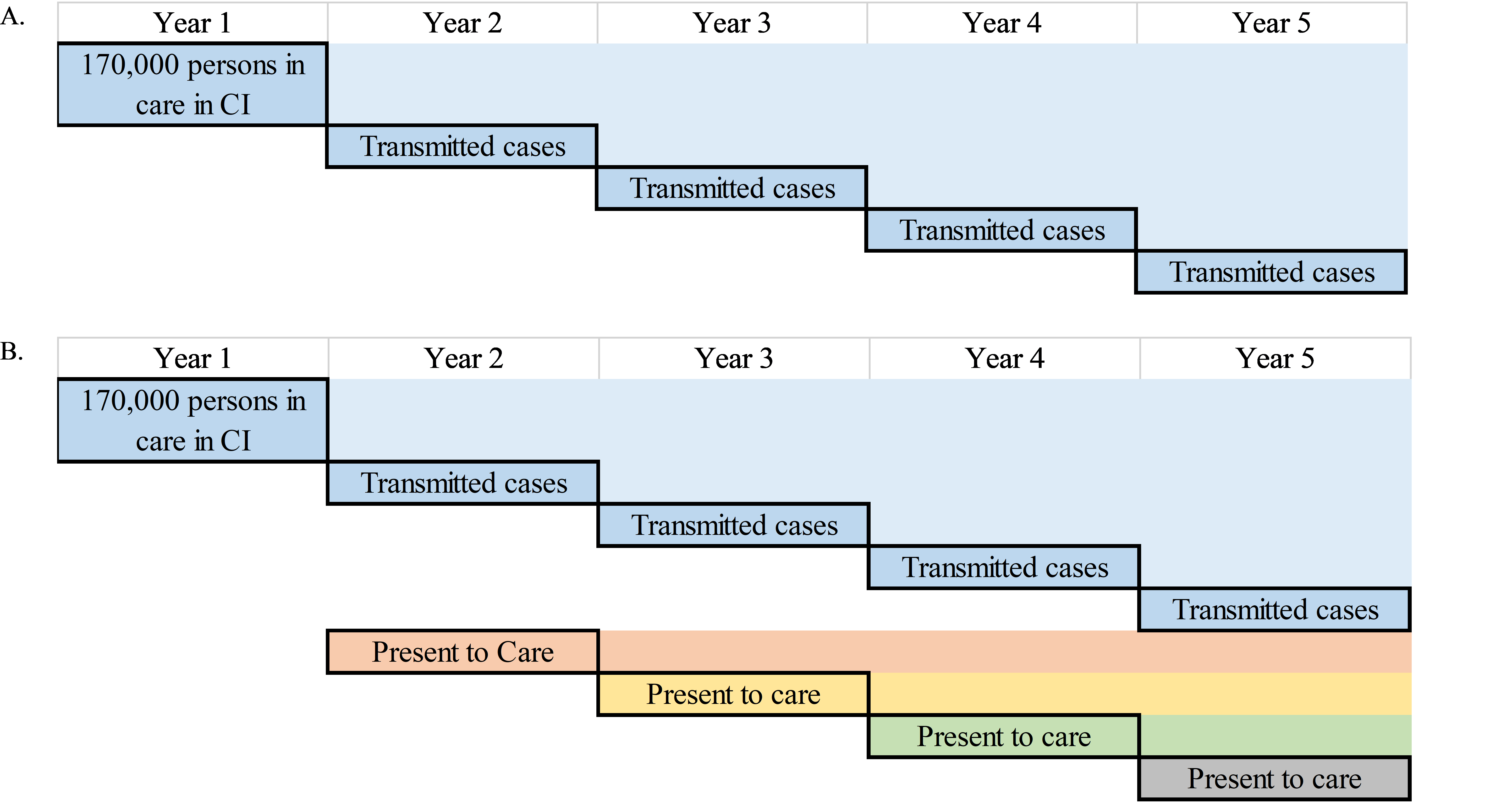

Supplement: S1 Fig — The populations included in the cost-effectiveness analysis (Panel A) were the 170,000 persons currently in care in Côte d’Ivoire (CI) as well as all transmitted cases arising from this population (including first generation and higher order transmissions). We restricted the population modeled to persons in care, and their transmitted cases, excluding persons with undiagnosed HIV, to isolate the effect of a policy change regarding ART initiation criteria. The populations modeled in the budget impact analysis (Panel B) included the 170,000 persons currently in care in Côte d’Ivoire and their transmitted cases, as well as persons newly presenting to care each year over the next five years. Each outlined box represents the entry of the specified cohort into the analysis. To estimate the number expected to enter care each year over the next 5 years (the “present to care” cohort), we began with historical data showing a yearly average of 14,000 people entering HIV care in Côte d’Ivoire [17]. For the Immediate ART and the ART<500/μL strategies, we subtracted from the 14,000 the number of transmissions prevented compared with the ART<350/μL strategy. Because the ART<350/μL cohort has the most transmissions of the strategies modeled, any transmissions averted by ART<500/μL and Immediate ART are excluded in the budget impact analysis for those strategies. We included an estimate of the costs of undiagnosed persons presenting to care in Côte d’Ivoire in the budget impact analysis to better project total HIV program costs under the different ART initiation thresholds. (TIF) [file pone.0219068.s001.tif]

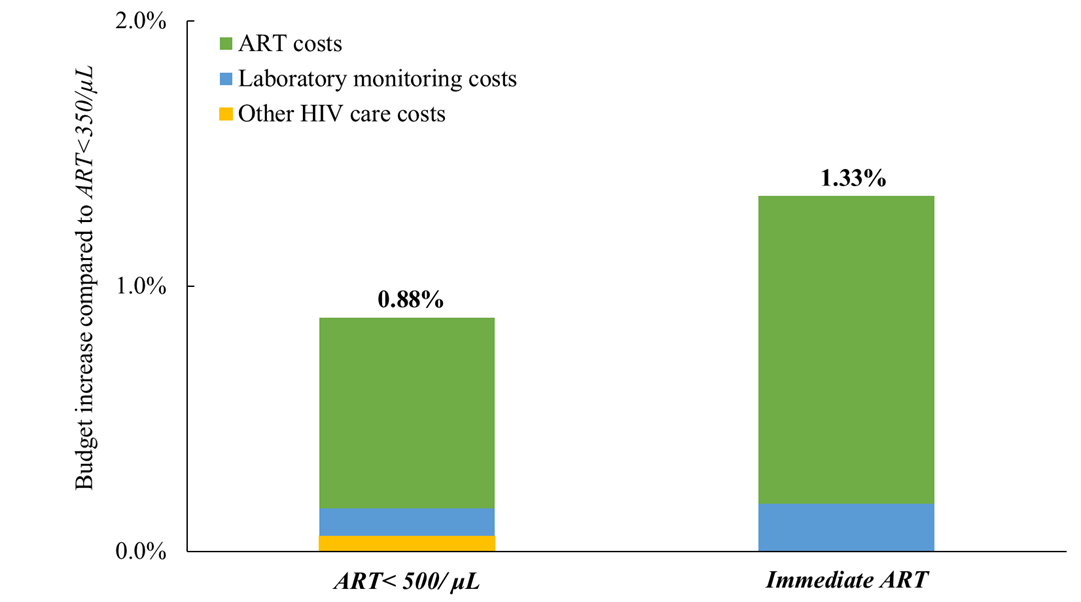

Supplement: S2 Fig — Each bar represents the 5-year proportional budget impact of ART<500/μL (left) and Immediate ART (right) compared to ART<350/μL. The height of the bars represents the impact on the total budget, measured in % budget increase compared to ART<350/μL. The change in other HIV care costs (orange), laboratory monitoring costs (blue), and ART costs (green) are also shown as a proportion of the change in total costs compared to ART<350/μL Most of the budget increases for ART<500/μL and Immediate ART at 5 years are in ART costs. ART: antiretroviral therapy. (TIF) [file pone.0219068.s002.tif]
