# Supplementary material for: Cost-effectiveness and budget impact of immediate antiretroviral therapy initiation for treatment of HIV infection in Côte d’Ivoire: A model-based analysis
Source: PLoS One. 2019 Jun 27;14(6):e0219068. doi: 10.1371/journal.pone.0219068 (PMC6597104; doi:10.1371/journal.pone.0219068)
Supplement: S1 Table — (DOCX) [file pone.0219068.s003.docx]

**S1 Table. Undiscounted clinical and economic outcomes of ART initiation according to CD4 threshold or immediate ART initiation in Côte d’Ivoire, corollary to Table 2**

1. **Transmissions and cost-effectiveness**

|  |  | **Life expectancy (years) ^†^** | **Transmissions caused, 10y** | **Total life-years, 10y** | **Total costs, 10y** | **ICER, 10y ($/YLS)** |
| --- | --- | --- | --- | --- | --- | --- |
| **Strategy** |  |  |  |  |  |  |
| ART<350/µL |  | 26.26 | 47,500 | 1,618,000 | 1,233,310,000 | - |
| ART<500/µL |  | 26.57 | 44,800 | 1,632,000 | 1,242,250,000 | Dominated* |
| Immediate ART |  | 26.66 | 43,000 | 1,638,000 | 1,245,470,000 | 630 |

**^†^**Life expectancy is reported from time at entry to care.

*Dominated: A strategy that is less cost-effective (higher ICER) than the next most costly option, and thus not an economically efficient use of resources.

Abbreviations: y: year; ICER: incremental cost-effectiveness ratio; ART: antiretroviral therapy; YLS: year of life saved
