# Supplementary material for: Cost-effectiveness and budget impact of immediate antiretroviral therapy initiation for treatment of HIV infection in Côte d’Ivoire: A model-based analysis
Source: PLoS One. 2019 Jun 27;14(6):e0219068. doi: 10.1371/journal.pone.0219068 (PMC6597104; doi:10.1371/journal.pone.0219068)
Supplement: S2 Table — (DOCX) [file pone.0219068.s004.docx]

**S2 Table.** **Sensitivity analysis of mean CD4 at diagnosis for incident cohorts in evaluation of clinical and economic outcomes of ART initiation according to CD4 threshold or immediate ART initiation in Côte d’Ivoire**.

|  | **Undiscounted outcomes** | | **Discounted* outcomes** | | | |
| --- | --- | --- | --- | --- | --- | --- |
|  | **Total life years, 10y** | **Total costs, 10y** | | **Total life years, 10y** | **Total costs,**  **10y** | **ICER ($/YLS)** |
| **Mean CD4 count at diagnosis for incident cases, /µL**  **(259; 221 to 599)** | | | | | | |
| **221/µL** |  | | | | | |
| ART<350/µL | 1,665,000 | 1,231,920,000 | | 1,419,000 | 1,057,770,000 | -- |
| ART<500/µL | 1,678,000 | 1,241,430,000 | | 1,430,000 | 1,066,070,000 | Dominated** |
| Immediate ART | 1,684,000 | 1,245,030,000 | | 1,435,000 | 1,069,360,000 | 740 |
| **399/µL** |  | | | | | |
| ART<350/µL | 1,671,000 | 1,271,760,000 | | 1,424,000 | 1,089,380,000 | -- |
| ART<500/µL | 1,686,000 | 1,279,460,000 | | 1,436,000 | 1,096,260,000 | Dominated** |
| Immediate ART | 1,689,000 | 1,280,540,000 | | 1,439,000 | 1,097,530,000 | 540 |
| **499/µL** |  | | | | | |
| ART<350/µL | 1,674,000 | 1,309,440,000 | | 1,426,000 | 1,119,640,000 | -- |
| ART<500/µL | 1,688,000 | 1,314,710,000 | | 1,438,000 | 1,124,550,000 | Dominated** |
| Immediate ART | 1,692,000 | 1,314,040,000 | | 1,441,000 | 1,124,430,000 | 320 |
| **599/µL** |  | | | | | |
| ART<350/µL | 1,672,000 | 1,342,240,000 | | 1,425,000 | 1,146,600,000 | -- |
| ART<500/µL | 1,687,000 | 1,344,130,000 | | 1,437,000 | 1,148,740,000 | Dominated** |
| Immediate ART | 1,691,000 | 1,340,750,000 | | 1,440,000 | 1,146,430,000 | Cost-saving |

y: year; ICER: incremental cost-effectiveness ratio; YLS: year of life saved.

*Outcomes are discounted at 3% per year.

**Dominated: A strategy that is less cost-effective (higher ICER) than the next most costly option, and thus not an economically efficient use of resources.
