# Supplementary material for: Cost-effectiveness and budget impact of immediate antiretroviral therapy initiation for treatment of HIV infection in Côte d’Ivoire: A model-based analysis
Source: PLoS One. 2019 Jun 27;14(6):e0219068. doi: 10.1371/journal.pone.0219068 (PMC6597104; doi:10.1371/journal.pone.0219068)
Supplement: S3 Table — (DOCX) [file pone.0219068.s005.docx]

**S3 Table. 15- and 20-year clinical and economic outcomes of ART initiation according to CD4 threshold or immediate ART initiation in Côte d’Ivoire**

| 1. **15-year outcomes** | | | | | |
| --- | --- | --- | --- | --- | --- |
|  | **Life expectancy (years) ^†^** | **Transmissions caused, 15y** | **Total life-years,**  **15y** | **Total costs,**  **15y** | **ICER, 15y ($/YLS)** |
| **Strategy** |  |  |  |  |  |
| ART<350/µL | 16.05 | 79,900 | 1,949,000 | 1,451,610,000 | -- |
| ART<500/µL | 16.22 | 76,900 | 1,977,000 | 1,461,860,000 | Dominated* |
| Immediate ART | 16.39 | 74,900 | 1,990,000 | 1,465,120,000 | 330 |
| 1. **20-year outcomes** | | | | | |
|  | **Life expectancy (years) ^†^** | **Transmissions caused, 20y** | **Total life-years,**  **20y** | **Total costs,**  **20y** | **ICER, 15y ($/YLS)** |
| **Strategy** |  |  |  |  |  |
| ART<350/µL | 16.05 | 114,600 | 2,452,000 | 1,791,990,000 | -- |
| ART<500/µL | 16.22 | 111,500 | 2,492,000 | 1,803,910,000 | Dominated* |
| Immediate ART | 16.39 | 109,300 | 2,512,000 | 1,807,220,000 | 250 |

y: year; ICER: incremental cost-effectiveness ratio; ART: antiretroviral therapy; YLS: year of life saved.

**^†^**Life expectancy is reported from time at entry to care.

‡Results are undiscounted by convention [[14](#_ENREF_14)]. The results are for the prevalent cohort of 170,000 people with HIV in care in Côte d’Ivoire, their transmitted cases, and the additional estimated number of persons entering HIV care each year.

*Dominated: A strategy that is less cost-effective (higher ICER) than the next most costly option, and thus not an economically efficient use of resources [[14](#_ENREF_14)]. The 15-year ICER for *ART<500/µL* compared to *ART<350/µL* is $370/YLS. The 20-year ICER for *ART<500/µL* compared to *ART<350/µL* is $300/YLS.
