# Supplementary material for: Cost-effectiveness and budget impact of immediate antiretroviral therapy initiation for treatment of HIV infection in Côte d’Ivoire: A model-based analysis
Source: PLoS One. 2019 Jun 27;14(6):e0219068. doi: 10.1371/journal.pone.0219068 (PMC6597104; doi:10.1371/journal.pone.0219068)
Supplement: S4 Table — (DOCX) [file pone.0219068.s006.docx]

**S4 Table 4. 5-year annual budget impact, 2017 USD, in millions**

| **Annual budget year** | **Strategy** | **ART costs (millions USD)** | **Lab monitoring costs**  **(millions USD)** | **Other HIV care costs (millions USD)** | **Total costs**  **(millions USD)** | **Increase in expenditures compared to ART<350/µL**  **(millions USD), (%)** |
| --- | --- | --- | --- | --- | --- | --- |
| 1 | ART<350/µL | 13.5 | 11.1 | 127.0 | 151.6 | -- |
|  | ART<500/µL | 15.5 | 11.9 | 126.7 | 154.1 | 2.5 (1.64) |
|  | Immediate ART | 17.1 | 12.4 | 126.5 | 156.0 | 4.4 (2.87) |
| 2 | ART<350/µL | 16.5 | 7.5 | 129.6 | 153.6 | -- |
|  | ART<500/µL | 17.8 | 7.5 | 129.5 | 154.8 | 1.2 (0.81) |
|  | Immediate ART | 18.6 | 7.5 | 129.4 | 155.5 | 1.9 (1.22) |
| 3 | ART<350/µL | 17.1 | 7.1 | 134.5 | 158.8 | -- |
|  | ART<500/µL | 18.0 | 7.1 | 134.6 | 159.8 | 1.0 (0.64) |
|  | Immediate ART | 18.5 | 7.1 | 134.6 | 160.2 | 1.4 (0.91) |
| 4 | ART<350/µL | 18.6 | 7.1 | 139.8 | 165.4 | -- |
|  | ART<500/µL | 19.3 | 7.1 | 140.1 | 166.5 | 1.1 (0.66) |
|  | Immediate ART | 19.7 | 7.1 | 140.0 | 166.9 | 1.5 (0.88) |
| 5 | ART<350/µL | 19.8 | 7.3 | 145.4 | 172.5 | -- |
|  | ART<500/µL | 20.5 | 7.4 | 145.9 | 173.7 | 1.2 (0.70) |
|  | Immediate ART | 20.9 | 7.4 | 145.8 | 174.1 | 1.6 (0.89) |

ART: antiretroviral therapy

Results are undiscounted by convention [[14](#_ENREF_14)]. The results are for the prevalent cohort of 170,000 people with HIV in care in Côte d’Ivoire, their transmitted cases, and the additional estimated number of persons entering HIV care each year.
